# Predicting mortality in critically ill patients requiring renal replacement therapy for acute kidney injury in a retrospective single-center study of two cohorts

**DOI:** 10.1038/s41598-022-14497-z

**Published:** 2022-06-17

**Authors:** Mikko J. Järvisalo, Noora Kartiosuo, Tapio Hellman, Panu Uusalo

**Affiliations:** 1 Kidney Center, Turku University Hospital and University of Turku, Turku, Finland; 2 Department of Anaesthesiology and Intensive Care, University of Turku, Turku, Finland; 3 Perioperative Services, Intensive Care and Pain Medicine, Turku University Hospital, Turku, Finland; 4 Centre for Population Health Research, University of Turku and Turku University Hospital, Turku, Finland; 5 Research Centre of Applied and Preventive Cardiovascular Medicine, University of Turku, Turku, Finland; 6 Department of Mathematics and Statistics, University of Turku, Turku, Finland; 7 Intensive Care Unit, Turku University Hospital, Building 18, TG3B, Hämeentie 11, 20520 Turku, Finland

**Keywords:** Continuous renal replacement therapy, Haemodialysis

## Abstract

Half of the critically ill patients with renal replacement therapy (RRT) dependent acute kidney injury (AKI) die within one year despite RRT. General intensive care prediction models perform inadequately in AKI. Predictive models for mortality would be an invaluable complementary tool to aid clinical decision making. We aimed to develop and validate new prediction models for intensive care unit (ICU) and hospital mortality customized for patients with RRT dependent AKI in a retrospective single-center study. The models were first developed in a cohort of 471 critically ill patients with continuous RRT (CRRT) and then validated in a cohort of 193 critically ill patients with intermittent hemodialysis (IHD) as the primary modality for RRT. Forty-two risk factors for mortality were examined at ICU admission and CRRT initiation, respectively, in the first univariate models followed by multivariable model development. Receiver operating characteristics curve analyses were conducted to estimate the area under the curve (AUC), to measure discriminative capacity of the models for mortality. AUCs of the respective models ranged between 0.76 and 0.83 in the CRRT model development cohort, thereby showing acceptable to excellent predictive power for the mortality events (ICU mortality and hospital mortality). The models showed acceptable external validity in a validation cohort of IHD patients. In the IHD validation cohort the AUCs of the MALEDICT RRT initiation model were 0.74 and 0.77 for ICU and hospital mortality, respectively. The MALEDICT model shows promise for mortality prediction in critically ill patients with RRT dependent AKI. After further validation, the model might serve as an additional clinical tool for estimating individual mortality risk at the time of RRT initiation.

## Introduction

One-year mortality in critically ill patients with acute kidney injury (AKI) requiring continuous renal replacement therapy (CRRT) exceeds 50% and has not improved in spite of developments in the field of intensive care during the last decade^1,2^.

Previous studies have indicated that scores used to assess severity of disease in intensive care patients, such as, Acute Physiology and Chronic Health Evaluation (APACHE)^3–5^, Simplified Acute Physiology Score (SAPS)^6^ and Sequential Organ Failure assessment score (SOFA)^5,7^ measured at the time of intensive care unit (ICU) admission or CRRT initiation, are associated with mortality in critically ill AKI patients but nevertheless perform inadequately in predicting mortality^8,9^.

Renal failure with a need for renal replacement therapy (RRT) substantially increase healthcare resource requirements and lead to increased costs and duration of hospital stay^10,11^. In spite of RRT half of the dialyzed ICU patients with AKI die within 1 year. More precise predictive models for mortality would be an invaluable complementary tool to aid clinical decision making in this highly morbid patient group and to recognize the patients that are likely to benefit from an increase in the intensity of care by RRT initiation. Previous available data have suggested that a combination of clinical evaluation and a predictive model risk estimate may improve the detection of patients with high or low survival probability compared to mere clinical judgment^12^.

Several observational studies have aimed to validate prediction models including conventional intensive care scoring systems or new models including machine learning based algorithms and the Mortality Scoring system for AKI with CRRT (MOSAIC) score^13^ for mortality in critically ill AKI patients requiring RRT. Most studies have examined risk factor effects assessed at a single time point during intensive care and the performance and external validation of the prediction models has been limited.

Therefore, we aimed to develop and validate respective predictive models for ICU and hospital mortality in critically ill patients with AKI requiring CRRT in a retrospective cohort, using data available on ICU admission and at CRRT initiation. Furthermore, the external validity of the models was examined in an independent cohort of critically ill AKI patients with intermittent hemodialysis (IHD) as the primary modality for RRT.

## Materials and methods

This retrospective cohort study consecutively included all patients admitted to the ICU of the Turku University Hospital requiring continuous veno-venous hemodialysis (CVVHD) (493 participants) as a primary modality for RRT between January 1 2010 and December 31 2019. As the focus of this pre-specified report was to develop and validate respective predictive models for ICU and hospital mortality in critically ill patients with AKI requiring RRT, all patients on maintenance dialysis (22 patients) were excluded from the study. No power calculations were performed due to the retrospective setting of the study. After model development in CRRT patients, we studied the external validity of the developed models in critically ill AKI patients that were started on IHD (not CRRT) as a primary RRT modality during the same time period at our ICU (240 patients: 47 patients with previous maintenance dialysis dependency were excluded leaving 193 patients for the validation analyses).

All data on patient demographics, disease history and medications at baseline were manually collected from the electronic patient records of the research hospital. Biochemical data at ICU admission as well as cumulative data on RRT and ICU clinical parameters were extracted from the clinical information software of the research ICU. All patients were followed-up from the electronic patient records to collect outcome data on ICU and hospital mortality.

Immunosuppression was defined as ongoing use of immunosuppressive medication: corticosteroids (methylprednisolone dose (or equivalent dose) exceeding 10 mg/day), calcineurin inhibitors, mycophenolate mofetil, azathioprine, immunosuppressive monoclonal antibodies used for active autoimmune disorders or cytotoxic chemotherapy agents administered within 1 year.

### CVVHD and IHD

CVVHD for all patients receiving CRRT was performed according to a standard protocol employed in our centre using Fresenius Multifiltrate CRRT monitors and 1.80 m^2^ polysulfone hemofilter Ultraflux AV1000 or Ultraflux EMiC2 membranes with the CiCa^®^ dialysate K2 and 4% trisodium citrate to achieve regional citrate anticoagulation (Fresenius Medical Care, Bad Hamburg, Germany). Blood and dialysate flow rates were prescribed according to the weight of the patient and by the caring ICU physician to target a dialysis dose of 30 ml/kg/h. The methodology for CRRT remained unaltered for the entire study period and CVVHD was the modality for each patient in the study.

IHD was performed using Fresenius Cordiax 5008 dialysis monitors with 2.5–5 h treatment duration, blood flow rates 170–300 ml/min, dialysate flow rate of 500 ml/min, and low-molecular-weight heparin (LMWH) anticoagulation dependent on the clinical condition of the patient and the running number of IHD treatment.

In Finland CRRT is the primary modality in approximately 70% of all RRT initiations in ICUs as CRRT is considered the modality of choice for hemodynamically unstable patients with the highest severity of illness^14^.

### Ethics

The study protocol was approved by the Turku University Clinical Research Center scientific review board (Turku CRC) and the Hospital district of Southwest Finland (Reference number: T143/2019). The patient identity numbers were removed and the hospital software data combined before the statistical analyses. For this retrospective, register-based, non-interventional study the Turku CRC and Hospital district of Southwest Finland joint review board waived the need for informed consent in terms of data collection and analysis and publication of results. All experiments were performed in accordance with relevant (STROBE) guidelines and regulations and all methods were carried out in accordance with relevant guidelines and regulations including the Helsinki declaration.

### Statistics

Results are reported as mean ± standard deviation (SD) for normally distributed covariates and as median (inter-quartile range (IQR)) for skewed covariates unless stated otherwise. Categorical variables are presented with absolute and relative (percentage) frequencies. For the skewed variables, we examined different transformations (log_e_-transformation, square root transformation and square transformation) to normalize distributions, and the best transformation for each variable was chosen according to visual examination or tests for normality (Shapiro–Wilk, Kolmogorov–Smirnov). Student’s t-test was used to compare continuous normally distributed covariates and Chi-square test for categorical covariates in the study subgroups. For skewed variables without suitable transformations the comparisons between groups were done using a non-parametric Kruskall–Wallis test.

The univariate associations between the dependent variables and tested covariates were initially explored by entering the covariates separately into logistic regression models. A total of 42 different covariates were first examined using respective univariate models during model development. Risk factors included in the first univariate models are listed in Supplementary Table 1. All continuous variables included in the models were measured at both ICU admission and CRRT initiation. Covariates with univariate associations with the dependent variable at p ≤ 0.05 significance level were included in the multivariable logistic regression models. Multiple imputation with 25 imputations was performed for each response (ICU mortality and hospital mortality, respectively) and at both measurement time points (ICU admission and CRRT initiation, respectively) for those variables with p < 0.05 in the univariate models. Then, in each imputed data set, these variables were entered in a multivariable model with stepwise selection. Prior to using the stepwise selection procedure, we removed variables that described the same phenomena and caused multicollinearity. Multicollinearity was assessed by examining variance inflation factors at several stages of the analyses.

A variable was chosen in the final model if the selection procedure chose the variable to the models in over 50%, i.e. ≥ 13 imputations. The results of the final model were combined over the multiple imputed data sets. Hosmer–Lemeshow goodness-of-fit test was performed for the multivariable models to assess the goodness of fit of the models.

Receiver operating characteristics (ROC) curve analyses were conducted to estimate the area under the curve (AUC) as a measure of discriminative capacity of the multivariable models for different mortality events, respectively. The ROCCONTRAST function of SAS was employed to compare AUCs (discriminative capacity) between the generated final multivariable models and traditional intensive care scoring systems (APACHE, SAPS and SOFA) at ICU admission and CRRT initiation, respectively, for the mortality events. Generally, we consider an AUC > 0.90 outstanding, an AUC 0.80–0.90 excellent, an AUC 0.70–0.80 acceptable and an AUC < 0.70 poor discrimination.

Finally, the external validity of the models developed in the CRRT population was examined in an independent cohort of 193 critically ill AKI patients with IHD as the first modality for RRT. Using the same selected variables of the models developed in the CRRT cohort, respective risk assessment equations for the IHD cohort were developed for further validation. However, as the mortality was significantly lower in the IHD population compared to the CRRT population the coefficients in these IHD specific equations differed from the CRRT cohort equations. Furthermore, we also aimed to validate the use of the equations developed in the CRRT cohort directly in the IHD cohort, without altering the models.

All statistical analyses were performed using statistical analysis system, SAS version 9.3 (SAS Institute Inc., Cary NC). p < 0.05 was considered statistically significant and all tests were two-sided.

## Results

The CRRT study population included 471 CRRT patients (138/29% women) with a mean age of 64.8 ± 13.1 years. ICU mortality was 34.2% and hospital mortality 42.3%. The characteristics of the CRRT study population and comparisons between hospital survivors and non-survivors are shown in Table 1. In the CRRT cohort 396 / 471 patients (84.1%) had data on baseline eGFR (within one year of admission). A total of 149 patients had prior CKD (eGFR < 60 ml/min/1.73 m^2^). CKD was not significantly associated with ICU (OR 1.251, 95% CI 0.839–1.867, p = 0.27) or hospital (OR 1.348, 95% CI 0.916–1.984, p = 0.13) mortality and therefore not included in the final models. The median time to dialysis initiation from ICU admission was 11 (3–31) h in the CRRT patients and, significantly longer, 19 (5–50) h in the IHD cohort (p = 0.001). At the time of RRT initiation 83 (18%) patients had hyperkalemia > 5.5 mmol/l, 173 (37%) patients Urea ≥ 20 mmol/l, 182 (39%) patients pH < 7.20 and 130 (28%) patients a cumulative fluid balance exceeding 5% of their body weight in the CRRT cohort. In the IHD cohort 38 (20%) patients had hyperkalemia > 5.5 mmol/l, 96 (50%) patients had Urea ≥ 20 mmol/l, 27 (14%) patients had pH < 7.20 and 32 (17%) patients had fluid overload.

**Table 1:** Characteristics of the study patients.

	All	Hospital survivors	Non-survivors	P-value
Number of subjects	471	272	199	–
Women [n, (%)]	138 (29)	79 (29)	59 (30)	0.89
Age (years)	66.4 (58.1–73.9)	64.9 (55.4–71.6)	68.6 (61.3–76.4)	**0.0003**
Surgical patients [n, (%)]	175 (37)	88 (32)	87 (44)	**0.01**
Hypertension [n, (%)]	304 (65)	173 (64)	131 (66)	0.62
Diabetes [n, (%)]	189 (40)	111 (41)	78 (39)	0.72
Heart failure [n, (%)]	113 (24)	64 (24)	49 (25)	0.78
Coronary artery disease [n, (%)]	134 (28)	63 (23)	71 (36)	**0.003**
Cerebrovascular disease [n, (%)]	47 (10)	26 (10)	21 (11)	0.72
Peripheral arterial disease [n, (%)]	60 (13)	29 (11)	31 (16)	0.11
Solid malignancy [n, (%)]	35 (7)	17 (6)	18 (9)	0.25
Pulmonary disease [n, (%)]	69 (15)	39 (14)	30 (15)	0.82
Liver cirrhosis [n, (%)]	15 (3)	6 (2)	9 (5)	0.16
Immunosuppression [n, (%)]	61 (13)	27 (10)	34 (17)	**0.02**
Sepsis [n, (%)]	245 (52)	132 (49)	113 (57)	0.08
Mechanical ventilation [n, (%)]	348 (74)	173 (64)	175 (88)	** < 0.0001**
Vasopressor use at ICU admission [n, (%)]	386 (82)	217 (80)	169 (85)	0.15
SOFA score at ICU admission	10 (7–12)	10 (7–12)	11 (7–13)	**0.004**
SOFA score at CRRT initiation	12 (9–14)	11 (9–13)	13 (11–15)	** < 0.0001**
APACHE score at ICU admission	25 (21–30)	24 (20–29)	27 (22–32)	**0.0002**
APACHE score at CRRT initiation	26 (21–31)	25 (21–30)	27 (23–32)	**0.0002**
SAPS score at ICU admission	55 (45–66)	52 (42–63)	60 (50–71)	** < 0.0001**
SAPS score at CRRT initiation	56 (46–66)	52 (43–63)	61 (51–71)	** < 0.0001**
Noradrenalin at ICU admission (µg/kg/min)	0.08 (0.02–0.16)	0.07 (0.02–0.15)	0.10 (0.03–0.18)	**0.01**
Noradrenalin at CRRT initiation (µg/kg/min)	0.13 (0.04–0.22)	0.10 (0.02–0.17)	0.17 (0.08–0.28)	** < 0.0001**
Hourly diuresis at ICU admission (ml/h)	14.6 (4.0–44.2)	18.7 (6.2–45.7)	8.5 (2.1–42.4)	** < 0.0001**
Hourly diuresis at CRRT initiation (ml/h)	10.2 (3.3–31.0)	14.9 (5.8–38.7)	5.8 (1.5–20.4)	** < 0.0001**
Mean arterial pressure at ICU admission (mmHg)	70 (60–82)	71 (61–84)	69 (59–81)	0.08
Mean arterial pressure at CRRT initiation (mmHg)	71 (63–81)	73 (64–84)	69 (62–77)	**0.0006**
Hemoglobin at ICU admission (g/l) *n* = 452	104 (93–120)	105 (95–121)	104 (91–119)	0.24
Hemoglobin at CRRT initiation (g/l) *n* = 460	103 (91–120)	104 (91–122)	102 (92–118)	0.37
Thrombocytes at ICU admission (10^9^/l) *n* = 452	134 (87–202)	144 (95–205)	122 (76–194)	**0.005**
Thrombocytes at CRRT initiation (10^9^/l) *n* = 459	135 (80–212)	160 (93–243)	103 (66–178)	** < 0.0001**
Bilirubin at ICU admission (µmol/l) *n* = 331	14 (8–32)	12 (7–26)	19 (9–43)	**0.0006**
Bilirubin at CRRT initiation (µmol/l) *n* = 357	14 (8–32)	11 (7–23)	20 (10–42)	** < 0.0001**
Creatinine at ICU admission (µmol/l) *n* = 421	216 (143–339)	243 (153–378)	190 (137–291)	**0.02**
Creatinine at CRRT initiation (µmol/l) *n* = 456	295 (190–415)	335 (217–490)	248 (171–357)	** < 0.0001**
Urea at ICU admission (mmol/l) *n* = 340	14.0 (8.5–23.1)	13.7 (8.9–22.1)	14.7 (8.3–23.5)	0.91
Urea at CRRT initiation (mmol/l) *n* = 371	19.0 (11.9–27.6)	20.0 (12.6–27.8)	17.6 (10.5–27.1)	0.13
Potassium at ICU admission (mmol/l) *n* = 464	4.4 (3.9–4.9)	4.3 (3.9–4.9)	4.4 (3.9–5.0)	0.41
Potassium at CRRT initiation (mmol/l) *n* = 392	4.2 (3.8–4.6)	4.1 (3.7–4.5)	4.3 (3.9–4.8)	**0.0004**
pH at ICU admission *n* = 465	7.27 (7.19–7.34)	7.28 (7.20–7.35)	7.26 (7.18–7.33)	**0.01**
pH at CRRT initiation *n* = 391	7.28 (7.19–7.36)	7.31 (7.22–7.37)	7.26 (7.12–7.34)	** < 0.0001**
Bicarbonate at ICU admission (mmol/l) *n* = 464	17.2 ± 4.8	17.6 ± 4.8	16.7 ± 4.7	**0.02**
Bicarbonate at CRRT initiation (mmol/l) *n* = 390	17.7 (14.4–20.8)	19.0 (15.4–21.2)	16.6 (12.9–19.4)	** < 0.0001**
Lactate at ICU admission (mmol/l) *n* = 463	3.2 (1.7–7.1)	2.4 (1.3–5.7)	4.8 (2.2–9.3)	** < 0.0001**
Lactate at CRRT initiation (mmol/l) *n* = 388	2.4 (1.2–7.3)	1.7 (1.0–4.3)	4.7 (2.0–9.2)	** < 0.0001**

Categorical values in parentheses are % unless stated otherwise. Continuous variables are expressed as mean (± SD) or median (IQR) for normally distributed and skewed covariates, respectively.

Hypertension was defined as a clinical diagnosis of hypertension and cerebrovascular disease as a diagnosis of ischemic or hemorrhagic stroke or transient ischemic attack observed in the patient records, respectively. Heart failure was defined as a clinical diagnosis of congestive heart failure observed in the patient records or ejection fraction < 50% or diastolic heart failure observed in echocardiography. Pulmonary disease was denoted as a prior diagnosis of asthma, chronic obstructive pulmonary disease or chronic interstitial lung disease. Coronary artery disease and peripheral arterial disease were defined as previously diagnosed conditions.

*ICU* intensive care unit, *SOFA-score* Sequential Organ Failure Assessment score, *CRRT* continuous renal replacement therapy, *APACHE* Acute Physiology and Chronic Health Evaluation II score, *SAPS* Simplified Acute Physiology II score.

Significant values are in bold.

In the CRRT population a total of 191 patients were dialysis dependent at the time of death (n = 168) or hospital discharge (n = 23). During the study period a total of 17,316 patients were admitted to the research ICU leading to an observed incidence of RRT of 3.8%.

**Figure 1:**
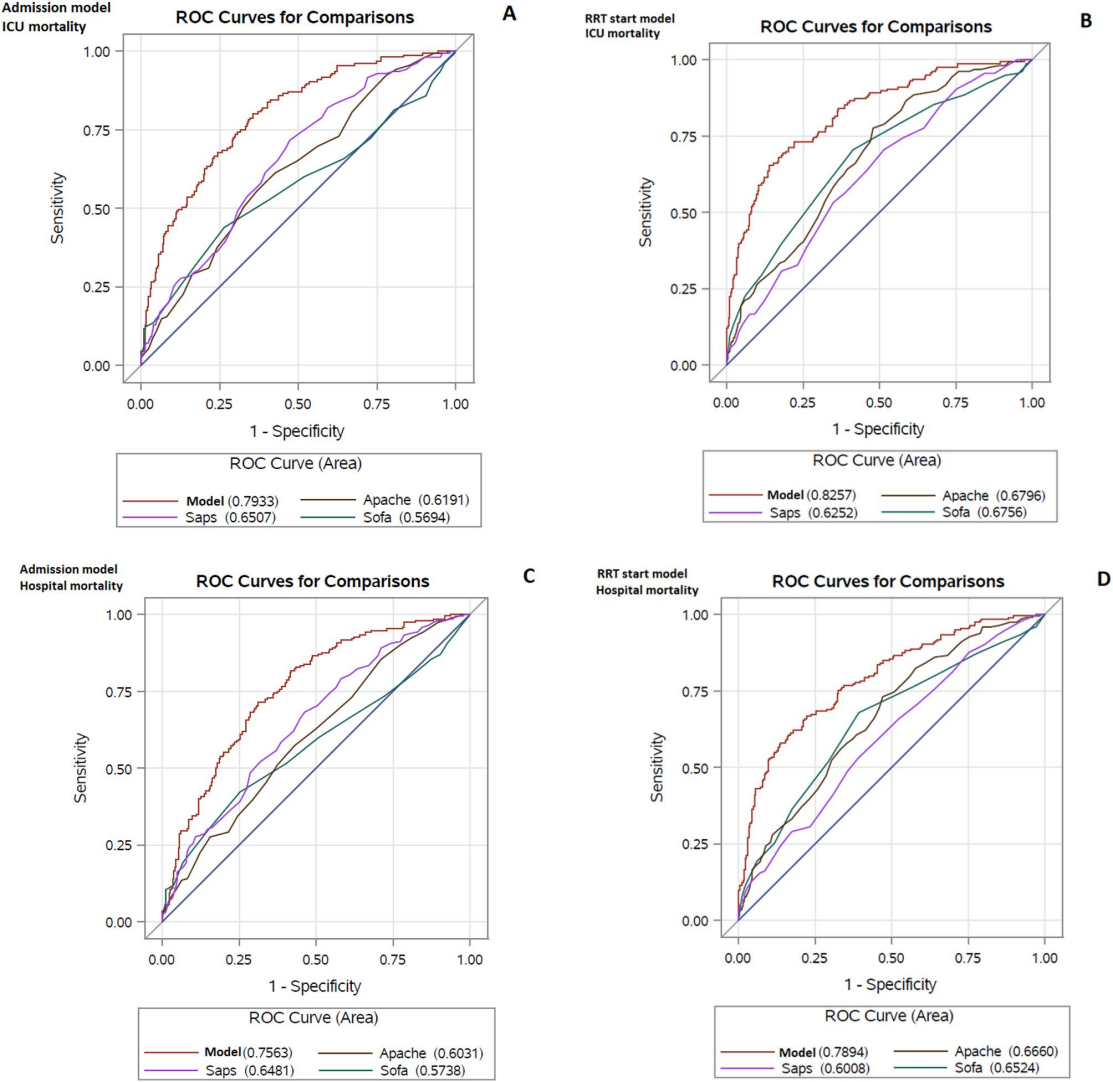
Receiver operating characteristics (ROC) curves for the final multivariable models for ICU and hospital mortality in the CRRT model development cohort. (**A**) ICU admission model for ICU mortality; (**B**) the MALEDICT RRT initiation model for ICU mortality; (**C**) ICU admission model for hospital mortality and (**D**) the MALEDICT RRT initiation model for hospital mortality.

The final best ICU admission and RRT initiation (later MALEDICT) multivariable models for ICU and hospital mortality, respectively, are shown in Table 2. The AUCs of the respective models ranged between 0.76 and 0.83, thereby showing acceptable to excellent predictive power for the mortality events (ICU mortality and hospital mortality). The AUCs were somewhat higher for RRT initiation models for predicting both ICU and hospital mortality, respectively, compared to ICU admission models. All the final multivariable models showed significantly higher predictive power compared to SOFA, APACHE-II and SAPS-II scores (p < 0.0001 for all comparisons) (Fig. 1). The performance of SOFA, APACHE-II and SAPS-II scores measured at both ICU admission and CRRT initiation and MOSAIC score measured at CRRT initiation was poor for mortality risk prediction with AUCs for discrimination of patients deceased in the ICU or during hospital care, respectively, ranging between 0.57 and 0.68. The comparisons between the predictive power of the new developed risk estimate models versus APACHE-II, SAPS-II, SOFA and MOSAIC scores in the complete case CRRT population are shown in Table 3.

**Table 2:** Final multivariable models for ICU mortality and hospital mortality developed in the CRRT patient cohort.

ICU mortality		Hospital mortality	
ICU admission model	The MALEDICT RRT initiation model	ICU admission model	The MALEDICT RRT initiation model
**AUC**			
0.79 (range 0.78–0.79)	0.83 (range 0.81–0.84)	0.76 (range 0.75–0.77)	0.79 (range 0.78–0.80)
**Sensitivity**			
0.51	0.61	0.57	0.63
**Specificity**			
0.87	0.88	0.78	0.80
**Negative predictive value**			
0.77	0.81	0.71	0.75
**Positive predictive value**			
0.67	0.72	0.65	0.70
**Hosmer–Lemeshow**			
GOF 2.98, p = 0.92	GOF 9.70, p = 0.35	GOF 5.18, p = 0.73	GOF 6.42, p = 0.61
**Brier score**			
0.171	0.155	0.197	0.182
**Adjusted R square**			
0.31	0.39	0.26	0.33
**Misclassification rate**			
0.25	0.22	0.31	0.27
**Variables in model**			
Surgical patient	Age	Age	Age
Coronary artery disease	Coronary artery disease	Coronary artery disease	Coronary artery disease
Immunosuppression	Immunosuppression	Immunosuppression	Immunosuppression
Mechanical ventilation	Mechanical ventilation	Mechanical ventilation	Mechanical ventilation
Hourly diuresis at admission*	Hourly diuresis at CRRT start*	Hourly diuresis at admission*	Hourly diuresis at CRRT start*
Thrombocytes at admission*	Thrombocytes at CRRT start*	Bilirubin at admission*	Thrombocytes at CRRT start*
Lactate at admission*	Lactate at CRRT start*	Lactate at admission*	Lactate at CRRT start*
**Equations for risk assessment**			
Logit (ICU mortality risk) = − 1.324 + 0.603(Surgical patient) + 0.986(Immunosuppression) + 0.790 (Coronary artery disease) + 1.461(Mechanical ventilation)–0.348[log_e_ (Hourly diuresis)] − 0.435[log_e_ (Thrombocytes)] + 0.778[log_e_ (Lactate)]	Logit (ICU mortality risk) = − 1.473 + 1.565 (Mechanical ventilation) + 0.025 (Age) + 0.976 [log_e_ (Lactate)] − 0.375 [log_e_ (Hourly diuresis)] + 0.864 (Immunosuppression) + 0.683 (Coronary artery disease) − 0.639 [log_e_ (Thrombocytes)]	Logit (Hospital mortality risk) = − 4.350 + 0.027(Age) + 0.737 (Immunosuppression) + 0.503 (Coronary artery disease) + 1.442(Mechanical ventilation) − 0.265[log_e_(Hourly diuresis)] + 0.289[log_e_ (Bilirubin)] + 0.495[log_e_ (Lactate)]	Logit (Hospital mortality risk) = − 1.000 + 1.287 (Mechanical ventilation) + 0.031 (Age) + 0.711 [log_e_ (Lactate)] − 0.336 [log_e_ (Hourly diuresis)] + 0.678 (Immunosuppression) + 0.550 (Coronary artery disease) − 0.585 [log_e_ (Thrombocytes)]

*ICU* intensive care unit, *CRRT* continuous renal replacement therapy.

*log_e_-transformed.

**Table 3:** Comparisons between the predictive power of the new developed risk estimate models versus APACHE-II, SAPS-II, SOFA and MOSAIC scores in the complete case CRRT population.

ICU mortality				Hospital mortality			
Prediction model	AUC	Contrast estimate (95% CL)	P-value	Prediction model	AUC	Contrast estimate (95% CL)	P-value
**CRRT patients *n* = 462**							
ICU admission model	**0.79**			ICU admission model	**0.76**		
SOFA admission	0.57	− 0.22 (− 0.29 to − 0.16)	** < 0.0001**	SOFA admission	0.57	− 0.18 (− 0.25 to − 0.12)	** < 0.0001**
APACHE-II admission	0.62	− 0.17 (− 0.23 to − 0.11)	** < 0.0001**	APACHE-II admission	0.60	− 0.15 (− 0.22 to − 0.09)	** < 0.0001**
SAPS-II admission	0.65	− 0.14 (− 0.20 to − 0.09)	** < 0.0001**	SAPS-II admission	0.65	− 0.11 (− 0.16 to − 0.06)	** < 0.0001**
**CRRT patients *n* = 408**							
The MALEDICT RRT initiation model	**0.82**			The MALEDICT RRT initiation model	**0.78**		
SOFA RRT initiation	0.69	− 0.13 (− 0.19 to − 0.06)	**0.0001**	SOFA RRT initiation	0.67	− 0.11 (− 0.17 to − 0.05)	**0.0003**
APACHE-II RRT initiation	0.68	− 0.14 (− 0.19 to − 0.08)	** < 0.0001**	APACHE-II RRT initiation	0.68	− 0.10 (− 0.16 to − 0.05)	**0.0004**
SAPS-II RRT initiation	0.63	− 0.19 (− 0.26 to − 0.12)	** < 0.0001**	SAPS-II RRT initiation	0.61	− 0.17 (− 0.24 to − 0.10)	** < 0.0001**
MOSAIC RRT initiation	0.69	− 0.13 (− 0.19 to − 0.07)	** < 0.0001**	MOSAIC RRT initiation	0.63	− 0.15 (− 0.21 to − 0.09)	** < 0.0001**
**IHD patients *n* = 193**							
The best IHD ICU Admission model	**0.82**			**0.81**			
The best IHD initiation model	**0.80**			**0.81**			
The CRRT ICU Admission model	**0.78**			**0.80**			
The MALEDICT RRT initiation model	**0.74**			**0.77**			

Values for the ICU admission and RRT initiation models and comparisons between prediction models are mean values for 25 multiple imputations.

*CL* confidence limit, *ICU* intensive care unit, *SOFA-score* Sequential Organ Failure Assessment score, *APACHE* Acute Physiology and Chronic Health Evaluation II score, *SAPS* Simplified Acute Physiology II score, *CRRT* continuous renal replacement therapy, *RRT* renal replacement therapy.

Significant values are in bold.

There was only minor variability in the covariates included in the final best multivariable models. The ICU admission model for ICU mortality included the categorical variable for surgical patient group instead of age compared to the RRT initiation model. The ICU admission model for hospital mortality included bilirubin instead of thrombocytes compared to the respective RRT initiation model. When using the best variables at ICU admission for prediction of hospital mortality risk (Table 2) to predict ICU mortality (instead of hospital mortality) the risk prediction equation for ICU mortality was: Logit (ICU mortality risk) =  − 4.796 + 0.021 (Age) + 0.924 (Immunosuppression) + 0.623 (Coronary artery disease) + 1.770 (Mechanical ventilation) − 0.315 [loge (Hourly diuresis)] + 0.256 [loge (Bilirubin)] + 0.694 [loge (Lactate)]. The discriminative performance of this substitute model was similar to the best model shown in Table 2 [AUC 0.79 (0.78–0.80), p = 0.75].

When using the best variables at ICU admission for prediction of ICU mortality risk to predict hospital mortality (instead of ICU mortality) the risk prediction equation for ICU mortality was: Logit (hospital mortality risk) =  − 0.082 + 0.394 (Surgical patient) + 0.773 (Immunosuppression) + 0.715 (Coronary artery disease) + 1.194 (Mechanical ventilation) − 0.291 [loge (Hourly diuresis)] − 0.346 [loge (Thrombocytes)] + 0.522 [loge (Lactate)]. The discriminative performance of this substitute model was similar to the best model shown in Table 2 [AUC 0.75 (0.74–0.75), p = 0.29].

The RRT initiation models for predicting ICU and hospital mortality, however, were identical and included the need for Mechanical ventilation (yes/no), Age (years), LactatE (log_e_-transformed), hourly Diuresis (log_e_-transformed), Immunosuppression (yes/no), Coronary artery disease (yes/no) and Thrombocytes (log_e_-transformed) at RRT initiation as covariates (the MALEDICT model). The discrimination of the MALEDICT RRT initiation model for ICU mortality was excellent with a mean AUC in 25 consecutive imputations (range) of 0.83 (0.81–0.84). The model was well calibrated (Hosmer–Lemeshow goodness of fit 8.00, p = 0.35) and the Brier score was 0.15 (range 0.15–0.16). The model yielded a risk prediction equation for ICU mortality: Logit (ICU mortality risk) =  − 1.473 + 1.565 (Mechanical ventilation) + 0.025 (Age) + 0.976 [log_e_ (Lactate)] − 0.375 [log_e_ (Hourly diuresis)] + 0.864 (Immunosuppression) + 0.683 (Coronary artery disease) − 0.639 [log_e_ (Thrombocytes)] (Table 2).

**Figure 2:**
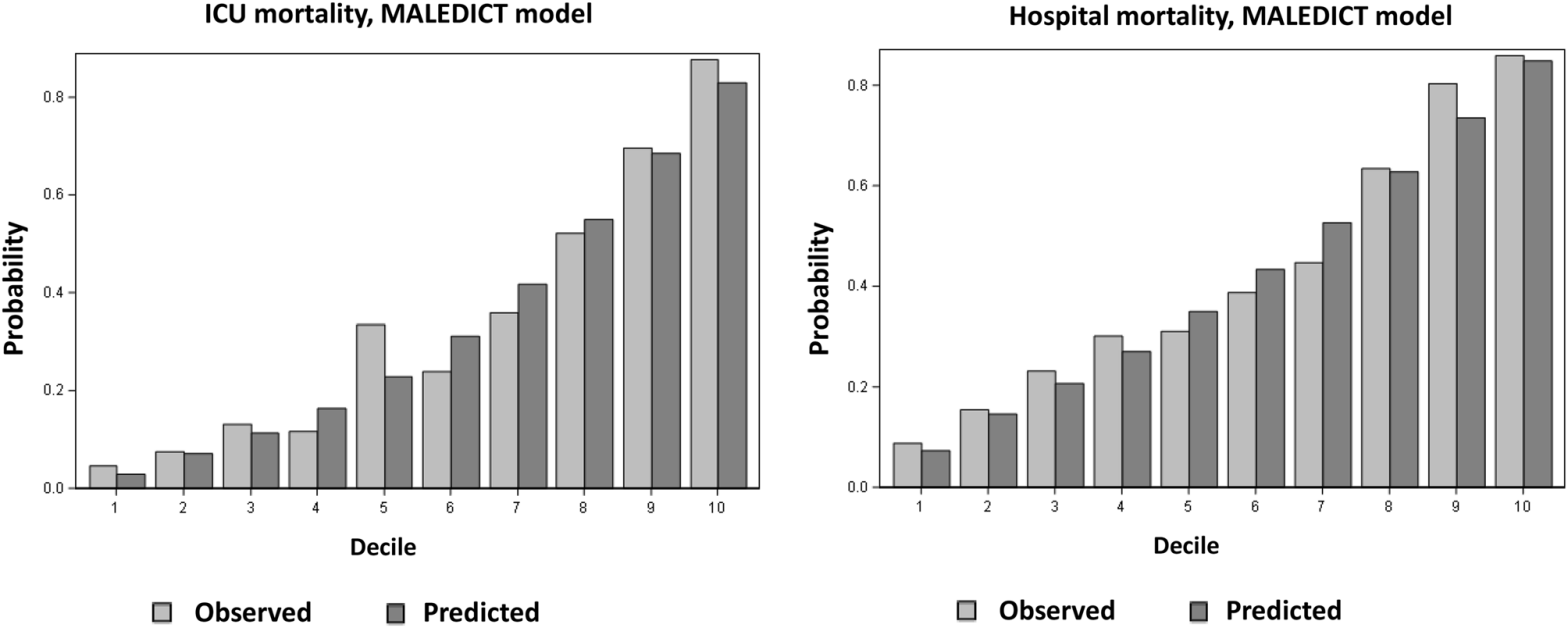
Calibration of the MALEDICT model for ICU and hospital mortality in the CRRT cohort. Average predicted probabilities of death and observed mortality according to decile of predicted probability of death.

The discrimination of the MALEDICT model for hospital mortality was acceptable with a mean AUC in 25 consecutive imputations (range) of 0.79 (0.78–0.80). The model was well calibrated (Hosmer–Lemeshow goodness of fit 8.00, p = 0.61) and the Brier score was 0.18 (range 0.18–0.19). The MALEDICT model yielded a risk prediction equation for hospital mortality: Logit (Hospital mortality risk) =  − 1.000 + 1.287 (Mechanical ventilation) + 0.031 (Age) + 0.711 [log_e_ (Lactate)] − 0.336 [log_e_ (Hourly diuresis)] + 0.678 (Immunosuppression) + 0.550 (Coronary artery disease) − 0.585 [loge (Thrombocytes)]. Figure 2 shows the calibration of the MALEDICT model for ICU and hospital mortality.

The characteristics of the IHD patient validation cohort are shown in Supplemental Table 2. Patients with IHD as the primary modality had significantly lower ICU and hospital mortality, severity of illness scores, lactate, bilirubin, vasopressor requirement and incidence of invasive mechanical ventilation and prevalence of coronary artery disease compared to the CRRT cohort whereas creatinine, thrombocytes and hourly diuresis were higher in IHD patients both at ICU admission and at the start of IHD. In spite of significant differences between the CRRT and the IHD cohorts, the predictive power of the models developed in CRRT patients and the best IHD models developed using the most significant variables in CRRT patients were, however, acceptable to excellent in both cohorts (Table 3).

The best IHD initiation model risk prediction equation for ICU mortality in IHD patients using the same variables as in the CRRT models was: Logit (ICU mortality risk) =  − 0.33 + 0.75 (Mechanical ventilation) − 0.01 (Age) + 0.69 [loge (Lactate)] − 0.34 [loge (Hourly diuresis)] + 0.63 (Immunosuppression) + 0.27 (Coronary artery disease) − 0.57 [loge (Thrombocytes)]. Correspondingly, the best IHD initiation model risk prediction equation for hospital mortality in IHD patients was: Logit (Hospital mortality risk) =  − 1.89 + 0.80 (Mechanical ventilation) + 0.04 (Age) + 0.38 [loge (Lactate)] − 0.08 [loge (Hourly diuresis)] + 0.678 (Immunosuppression) + 0.550 (Coronary artery disease) − 0.585 [loge (Thrombocytes)].

**Figure 3:**
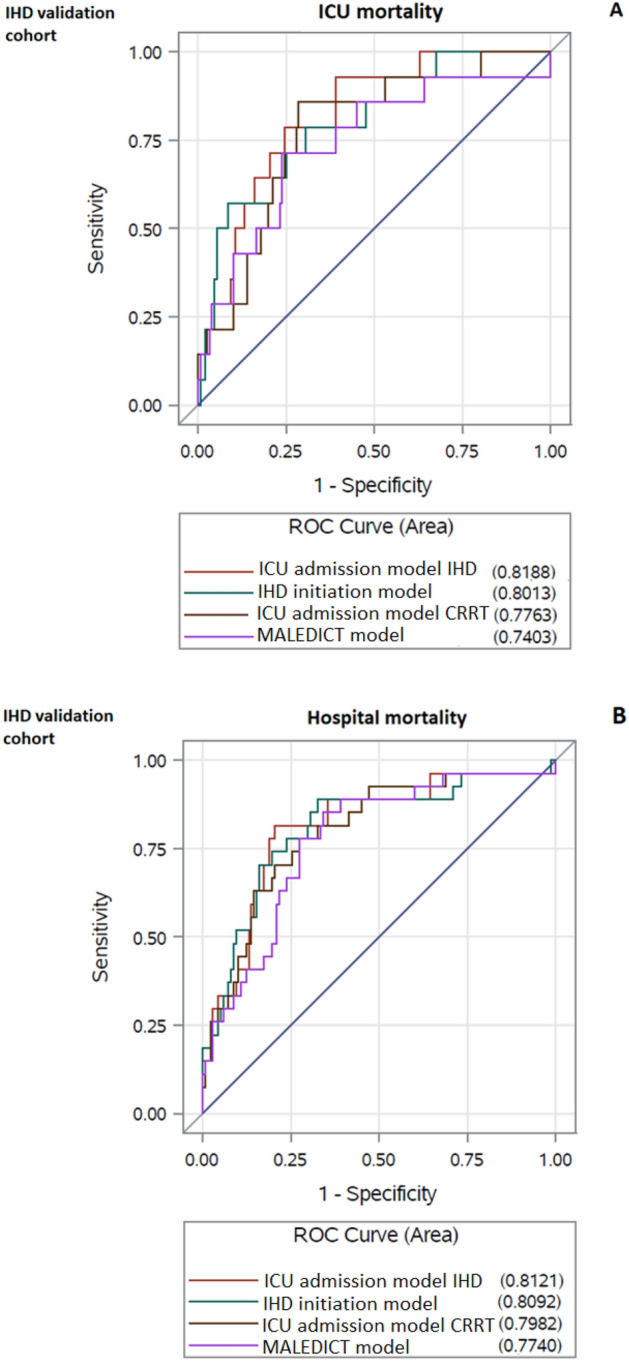
Receiver operating characteristics (ROC) curves for the final multivariable models for ICU and hospital mortality in the intermittent hemodialysis (IHD) validation cohort. (**A**) The best ICU admission and RRT initiation models for ICU mortality in IHD patients and the CRRT ICU admission and CRRT RRT initiation (MALEDICT) models in the IHD validation cohort; (**B**) the best ICU admission and RRT initiation models for hospital mortality in IHD patients and the CRRT ICU admission and CRRT RRT initiation (MALEDICT) models in the IHD validation cohort.

The AUCs of the best IHD ICU admission models (applying the same variables used in the CRRT models) were 0.82 (0.72–0.92) and 0.81 (0.72–0.91) and the AUCs of the best IHD initiation models were 0.80 (0.68–0.92) and 0.81 (0.71–0.91) for ICU and hospital mortality, respectively in the IHD validation cohort. The discrimination performances of the original CRRT ICU admission models in the IHD validation cohort were also acceptable and similar compared to the best IHD ICU admission model for ICU mortality [AUC 0.78 (0.66–0.90), p = 0.31] and hospital mortality [AUC 0.80 (0.70–0.89), p = 0.37]. The discrimination performance of the MALEDICT model in the IHD validation cohort was acceptable and similar compared to the best IHD initiation model for ICU mortality [AUC 0.74 (0.59–0.89, p = 0.24], and hospital mortality [AUC 0.77 (0.68–0.87), p = 0.20] (Fig. 3).

## Discussion

Our current results implicate that the novel predictive models developed in the present study using multiple laboratory, clinical and demographic data at both ICU admission and CRRT initiation, exceed the predictive power of traditional intensive care prognostic scores such as APACHE, SAPS and SOFA even when these scores, are recorded at both ICU admission and CRRT initiation, respectively. The predictive power of our new models for ICU mortality had higher AUCs compared to that recently reported using a machine learning algorithm^13^. Furthermore, our models showed acceptable external validity when applied to the ICU patients started on IHD as the primary modality for RRT during the same time period between 2010 and 2019.

It has been conclusively shown, that traditional intensive care prognostic scoring systems such as APACHE, SAPS and SOFA are of insufficient accuracy for predicting mortality in patients with AKI, especially in patients requiring RRT^15^. In critically ill patients AKI even without the need for RRT indisputably increases mortality as well as treatment costs and the length of hospital stay in survivors^10,11,16^. Furthermore, even the ICU survivors with AKI show an increased risk for developing chronic kidney disease (CKD) leading to a continuous increased need for medical services and costs. The incidence of RRT dependent AKI and associated mortality has remained high and unaffected by advances in intensive care medicine during recent years potentially due to increasing age and comorbidity of the ICU patient population^17^. Therefore, it would be invaluable to be able to assess patients’ individual mortality risk more reliably to target treatment to those more likely to survive past ICU and hospital care and to avoid futile treatment efforts and individual unwarranted suffering related to continued high intensity care when prognosis is dismal. Some previous studies have suggested that combining physicians’ clinical estimates and a predictive model for mortality risk assessment may increase detection of patients with high or low survival probability compared to mere clinical judgment^12^.

During recent years some observational studies have assessed the validity and predictive power of prediction models for early mortality in critically ill AKI patients requiring CRRT including conventional intensive care scoring systems such as APACHE, SAPS and SOFA or new models including machine learning based algorithms^13,15,18^. Most studies have examined the significance of risk factors assessed at a single time point during intensive care and the performance and external validation of the prediction models has been limited. In a recent study Kim et al. developed a Mortality Scoring system for AKI with CRRT (MOSAIC) for one-week mortality in 828 critically ill patients undergoing CRRT. All model data was gathered at CRRT initiation and the model development was based on modifying the APACHE and SOFA scores and then combining seven of the variables with the highest predictive power to yield the MOSAIC score. The model was validated using an independent cohort of CRRT patients and yielded AUCs of 0.77 (0.74–0.81) and 0.77 (0.73–0.82) for the original and the validation cohort, respectively^18^. The MOSAIC score was also examined in a later study in addition to machine learning models and showed acceptable predictive power for ICU mortality although the AUC was somewhat lower 0.72 (0.68–0.77) compared to the original MOSAIC cohort. The machine learning models showed slightly higher AUCs ranging between 0.75 and 0.78 compared to the MOSAIC score^13^. In the present study cohort, the performance of the MOSAIC score was poor and comparable to SOFA, APACHE and SAPS scores. In another previous study da Hora Passos and coworkers developed the Hepatic failure, LactatE, NorepInephrine, medical Condition and Creatinine (HELENICC) score for 7-day mortality prediction in 186 septic AKI patients on CRRT, which, outperformed the general ICU scores with an AUC of 0.82 (0.76–0.88) but has not been validated in an independent cohort^15^. As opposed to these previous studies, we aimed to develop models for predicting hospital mortality in addition to short-term ICU mortality. Furthermore, we examined over forty different risk factors for mortality in the primary univariate models measured both at ICU admission and at CRRT initiation, and the final models included only significant multivariable predictors of mortality. Continuous variables were used in the models without classification. The AUC of the MALEDICT model for ICU mortality exceeded and the AUC for hospital mortality was similar to the AUCs of previously reported mortality prediction models for one-week mortality^13,15,18^.

Not surprisingly the models developed for clinical and laboratory parameters examined at CRRT initiation were more accurate in predicting incident mortality compared to models constructed using parameters measured on ICU admission. Models using data at the time of CRRT initiation are especially of clinical relevance in affected patients as they may aid the clinician in estimating whether or not the patient is likely to benefit from CRRT initiation. Surprisingly the AUC of the ICU admission model slightly exceeded the AUC of the MALEDICT model in the IHD validation cohort. The predictive power of the models for hospital mortality, showed acceptable predictive value although AUCs were inferior compared to models for ICU mortality. It is easy to comprehend that the models predicted ICU mortality more accurately than hospital mortality, as when the patient survives through ICU care and is discharged to the ward it is likely that long term medical conditions and overall health and fitness prior to ICU admission become more important for future prognosis compared to variables measured during intensive care. The models constructed in the current study therefore apply best for short to medium term mortality risk prediction in critically ill patients with RRT dependent AKI. All of the models had, significantly higher predictive value compared to the APACHE, SAPS and SOFA scores.

We aimed to validate the developed MALEDICT model in an independent patient cohort of critically AKI patients receiving IHD as the first RRT modality. Furthermore, we developed individual specific equations for mortality prediction in IHD patients using the same variables that were included in the CRRT models. The IHD patients differed markedly and significantly from the CRRT patients in terms of disease severity as well as ICU and hospital mortality. This selection of patients to different modalities of RRT in critical care is natural as CRRT is considered the modality of choice for hemodynamically unstable patients with the highest severity of illness. The number of patients in the IHD validation cohort was somewhat limited as CRRT is the primary modality in approximately 70% of all RRT initiations in ICUs in Finland^14^. Nevertheless, the predictive value of the models for mortality was acceptable to excellent in both the CRRT and the IHD cohorts.

The current study has all the limitations of an observational study. The retrospective single center study design may increase the possibility of residual confounders and limit the generalizability of the findings. However, the patients were extensively studied and we examined over forty variables in the primary univariate models for each mortality event, and time point respectively. The quality of data was good due to the electronic patients records used at our center and the missingness in the entire data set was low. Furthermore, we employed multiple imputation with 25 consecutive imputations for each model and the resulting models are based on a summary of all the imputations. Both internal and external validity of the models was assessed and found to be acceptable. As our models are however, based on retrospective data from a single center the new models need to be validated in a prospective study in other centers in the future.

Although the predictive value of the models was between acceptable and excellent according to AUCs of the models, it is worth to notice that even the in the MALEDICT model with the highest AUC of 0.83 for ICU mortality, the rate of misclassification was still considerable 22%. This emphasizes the additive role of the prediction models/equations and scores for clinical decision-making which should not be blindly based on mere prediction modelling as no model will exactly predict whether a single patient will decease or not.

In conclusion, the models developed in the present study, show promise for mortality prediction in critically ill patients with RRT dependent AKI. After further validation in another independent cohort the MALEDICT model might serve as an additional clinical tool for estimating individual mortality risk of affected patients at the time of CRRT initiation.

## Supplementary Information

Supplementary Table 1.

Supplementary Table 2.
