# Supplementary material for: Predicting mortality in critically ill patients requiring renal replacement therapy for acute kidney injury in a retrospective single-center study of two cohorts
Source: Sci Rep. 2022 Jun 17;12:10177. doi: 10.1038/s41598-022-14497-z (PMC9205979; doi:10.1038/s41598-022-14497-z)
Supplement: Supplementary file 1 — Supplementary Table 1. [file 41598_2022_14497_MOESM1_ESM.docx]

**Supplemental Table 1. Variables included in the univariate models at ICU admission and RRT initiation for incident mortality events (ICU mortality and hospital mortality).**

| **Demographics and comorbidities** |
| --- |
| Age |
| Gender |
| Patient group (Medical/Surgical) |
| Chronic kidney disease |
| Hypertension |
| Diabetes |
| Cardiac failure |
| Coronary artery disease |
| Cerebrovascular disease |
| Peripheral arterial disease |
| Solid malignancy |
| Pulmonary disease |
| Liver cirrhosis |
| Immunosupression |
| **Physical parameters and treatments** |
| Mechanical ventilation |
| Noradrenalin dose |
| Mean arterial pressure |
| Hourly diuresis |
| Fluid balance at RRT initiation |
| **Laboratory parameters** |
| Hemoglobin |
| Leukocytes |
| Thrombocytes |
| C-reactive protein |
| Bilirubin |
| Alanine aminotransferase |
| International normalized ratio |
| Creatinine |
| Urea |
| Troponin T |
| Pro-BNP |
| Sodium |
| Potassium |
| Magnesium |
| Phosphorus |
| Ionized calcium |
| Total calcium |
| Chloride |
| Glucose |
| pH |
| Bicarbonate |
| Base excess |
| paCO2 |
| paO2 |
| Lactate |
