# Supplementary material for: Predicting mortality in critically ill patients requiring renal replacement therapy for acute kidney injury in a retrospective single-center study of two cohorts
Source: Sci Rep. 2022 Jun 17;12:10177. doi: 10.1038/s41598-022-14497-z (PMC9205979; doi:10.1038/s41598-022-14497-z)
Supplement: Supplementary file 2 — Supplementary Table 2. [file 41598_2022_14497_MOESM2_ESM.docx]

**Supplemental Table 2. Characteristics of the intermittent hemodialysis validation cohort.**

| Number of subjects | 193 |
| --- | --- |
| Women [n, (%)] | 62 (32) |
| Age (years) | 64.9 (51.1-72.5) * |
| Surgical patients [n, (%)] | 50 (26) ** |
| Coronary artery disease [n, (%)] | 27 (14) *** |
| Immunosuppression [n, (%)] | 26 (13) |
| Mechanical ventilation [n, (%)] | 68 (35) *** |
| SOFA score at ICU admission | 7 (5-9) *** |
| APACHE score at ICU admission | 22 (18-28) *** |
| SAPS score at ICU admission | 44 (34-53) *** |
| Vasopressor use at ICU admission [n, (%)] | 54 (28) *** |
| Hourly diuresis at ICU admission (ml/h) | 50 (21-123) *** |
| Hourly diuresis at CRRT initiation (ml/h) | 60 (30-120) *** |
| Mean arterial pressure at ICU admission (mmHg) | 82 (68-96) *** |
| Thrombocytes at ICU admission (10^9^/l) | 201 (134-284) *** |
| Thrombocytes at CRRT initiation (10^9^/l) | 192 (129-277) *** |
| Bilirubin at ICU admission (µmol/l) | 9 (5-17) *** |
| Bilirubin at CRRT initiation (µmol/l) | 9 (5-16) *** |
| Creatinine at ICU admission (µmol/l) | 294 (135-605) *** |
| Creatinine at CRRT initiation (µmol/l) | 352 (187-580) * |
| Lactate at ICU admission (mmol/l) | 1.5 (0.7-4.1) *** |
| Lactate at CRRT initiation (mmol/l) | 1.2 (0.7-2.2) *** |
| ICU mortality [n, (%)] | 14 (7) *** |
| Hopitl mortality [n, (%)] | 33 (17) *** |

Categorical values in parentheses are % unless stated otherwise. Continuous variables are expressed as median (IQR).

ICU = intensive care unit; SOFA-score = Sequential Organ Failure Assessment score; CRRT = continuous renal replacement therapy; APACHE = Acute Physiology And Chronic Health Evaluation II score; SAPS = Simplified Acute Physiology II score.

*, p<0.05; **, p<0.01; ***, p<0.001 compared to the CRRT cohort.
